# Acute Noise Exposure Is Associated With Intrinsic Apoptosis in Murine Central Auditory Pathway

**DOI:** 10.3389/fnins.2018.00312

**Published:** 2018-05-09

**Authors:** Moritz Gröschel, Dietmar Basta, Arne Ernst, Birgit Mazurek, Agnieszka J. Szczepek

**Affiliations:** ^1^Department of Otolaryngology, Unfallkrankenhaus Berlin, Charité Medical School, Berlin, Germany; ^2^Tinnitus Center, Berlin Institute of Health, Charité-Universitätsmedizin Berlin, Corporate Member of Freie Universität Berlin, Humboldt-Universität zu Berlin, Berlin, Germany; ^3^Department of Otorhinolaryngology, Head and Neck Surgery, Berlin Institute of Health, Charité-Universitätsmedizin Berlin, Corporate Member of Freie Universität Berlin, Humboldt-Universität zu Berlin, Berlin, Germany

**Keywords:** noise-induced hearing loss, central auditory system, acute noise exposure, APAF1, BCL2A1A

## Abstract

Noise that is capable of inducing the hearing loss (NIHL) has a strong impact on the inner ear structures and causes early and most obvious pathophysiological changes in the auditory periphery. Several studies indicated that intrinsic apoptotic cell death mechanisms are the key factors inducing cellular degeneration immediately after noise exposure and are maintained for days or even weeks. In addition, studies demonstrated several changes in the central auditory system following noise exposure, consistent with early apoptosis-related pathologies. To clarify the underlying mechanisms, the present study focused on the noise-induced gene and protein expression of the pro-apoptotic protease activating factor-1 (APAF1) and the anti-apoptotic B-cell lymphoma 2 related protein a1a (BCL2A1A) in the cochlear nucleus (CN), inferior colliculus (IC) and auditory cortex (AC) of the murine central auditory pathway. The expression of *Bcl2a1a* mRNA was upregulated immediately after trauma in all tissues investigated, whereas the protein levels were significantly reduced at least in the auditory brainstem. Conversely, acute noise has decreased the expression of *Apaf1* gene along the auditory pathway. The changes in APAF1 protein level were not statistically significant. It is tempting to speculate that the acoustic overstimulation leads to mitochondrial dysfunction and induction of apoptosis by regulation of proapoptotic and antiapoptotic proteins. The inverse expression pattern on the mRNA level of both genes might reflect a protective response to decrease cellular damage. Our results indicate the immediate presence of intrinsic apoptosis following noise trauma. This, in turn, may significantly contribute to the development of central structural deficits. Auditory pathway-specific inhibition of intrinsic apoptosis could be a therapeutic approach for the treatment of acute (noise-induced) hearing loss to prevent irreversible neuronal injury in auditory brain structures and to avoid profound deficits in complex auditory processing.

## Introduction

Over 5% of the world's population (360 million people) has disabling hearing loss, i.e., they have a hearing loss of at least 40 dB (WHO, 2015). Exposure to noise is a major cause of hearing loss, particularly in the industrialized countries, and is thought to be responsible for several audiological symptoms including tinnitus or hyperacusis (Le et al., 2017; Liberman, 2017).

### Noise-induced structural changes in cochlea

A lot of research regarding noise-induced changes has been performed with a focus on physiological and anatomical properties of the auditory system, particularly in the auditory periphery. Noise-induced pathologies of the central auditory system were much less studied as the peripheral ones. Noise has a strong impact on inner ear structures and induces early and most obvious pathophysiological changes by directly damaging sensory tissue due to mechanical forces and the electrochemical hyperexcitation, followed by progressive degeneration extending toward primary auditory afferents (Pujol and Puel, 1999; Hu et al., 2002, 2006; Wang et al., 2002; Hirose and Liberman, 2003; Yang et al., 2004; Henderson et al., 2006; Weisz et al., 2009; Eskiizmir et al., 2011). Although necrosis was thought to be the major cause of cochlear cell death, it became evident that apoptotic pathways play as well a fundamental role (Saunders et al., 1985; Hu et al., 2000; Pirvola et al., 2000; Bohne et al., 2007). Several studies have investigated the mechanisms underlying cochlear pathologies, providing evidence that the acoustically induced, overwhelming production of reactive oxygen species (ROS) is the key factor immediately contributing to cellular degeneration after noise exposure (Hamernik et al., 1984; Hu et al., 2002; Yamashita et al., 2004a; Yang et al., 2004; Henderson et al., 2006). Such contribution was reported to last up to several weeks after the acoustic injury.

The gene classes, in which the early changes in the expression have been reported due to a loss of auditory function include immediate early genes, inflammatory and oxidative stress responses genes and the cell death pathway genes. The relation between the ROS production and the expressional activation of various genes seems to play a crucial role in this process and was suggested to be particularly involved in cochlear apoptosis through activation of several cell-death pathways (Van De Water et al., 2004; Gross et al., 2007; Mazurek et al., 2011; Dinh et al., 2015). The expression of pro- and anti-apoptotic genes in the peripheral auditory system (i.e., the cochlea) changes within a few hours or days after pharmacologically- or noise-induced insult. During the first hours following the otological damage, the intrinsic, mitochondrial apoptotic pathway seems to be particularly involved due to oxidative stress by generation of ROS (Ohlemiller et al., 1999; Henderson et al., 2006). Contribution of the intrinsic pathway was indicated by activation of specific caspases (Nicotera et al., 2003; Han et al., 2006; García-Berrocal et al., 2007) and by expressional regulation of genes belonging to the family of B-cell lymphoma 2 (*Bcl2*) genes (Hu et al., 2009; Furness, 2015) or other intrinsic apoptosis-related genes (Yamashita et al., 2004a,b; Kirkegaard et al., 2006; Ding et al., 2009). Similar expression pattern has been also shown to occur in the aging cochlea, contributing to age-related hearing loss (Alam et al., 2001; Nevado et al., 2006; Tadros et al., 2008; Someya et al., 2009; Falah et al., 2016).

### Central pathophysiology induced by hearing loss

Studies of the central auditory system following noise exposure have determined several alterations in neuronal properties, including spontaneous and evoked hyperactivity, increased excitatory and reduced inhibitory neurotransmission, and tonotopic reorganization (Kaltenbach et al., 1998; Rajan and Irvine, 1998; Komiya and Eggermont, 2000; Wallhäusser-Franke et al., 2003; Basta and Ernst, 2005; Holt et al., 2005; Gröschel et al., 2011, 2014). Moreover, acoustic or mechanical trauma to the auditory periphery was shown to induce cellular degeneration in the central auditory pathway including cell and tissue shrinkage, axon degeneration, and neuronal cell loss. Such damage was shown to be present after initial damage and to persist for several days or weeks (Jean-Baptiste and Morest, 1975; Benson et al., 1997; Kim et al., 1997, 2004; Aarnisalo et al., 2000; Sekiya et al., 2009, 2012). Others have pointed at the appearance of plasticity markers in auditory brain immediately following the cochlear injury (Michler and Illing, 2002; Gil-Loyzaga et al., 2010). From these studies, it became evident that the lower ascending pathway—particularly the auditory brainstem—is primarily affected by cochlear injury, and that the observed changes may fundamentally affect the auditory function.

In healthy mice, histological studies revealed that the noise exposure leads to a reduction of cell density in the key structures of the central auditory system, whereby the first arising changes are most prominent in the auditory brainstem and then occur subsequently and to a lesser extend in the thalamic and cortical areas (Basta et al., 2005; Gröschel et al., 2010). Immunohistochemistry has identified apoptosis as one of main factors responsible for the above changes. Moreover, the time course of cell death supported the histological findings (Coordes et al., 2012; Fröhlich et al., 2017). A few studies investigated changes in the gene expression of the auditory brain in response to hearing loss. An altered expression of immediate early genes and neuroplasticity markers seems to play a particular role in generation of hyperactive neuronal disorders by changing synaptic neurotransmission (Wallhäusser-Franke et al., 2003; Oh et al., 2007; Kraus et al., 2009; Manohar et al., 2016; Tan et al., 2016). Further, expression of pro- and anti-apoptotic genes belonging to the *Bcl2* family has been suggested to impair hippocampal cell proliferation during pharmacological induction of hearing loss and to possibly affecting pathophysiological processes in the inferior colliculus (Manohar et al., 2014). Long-term inflammatory responses seem to be present after noise-induced hearing loss in the cochlear nucleus (Manohar et al., 2016).

It still remains unclear to which extend the central auditory system can be affected by a noise exposure, and which signaling pathways play a major role during the pathological processes. Further, it is of high relevance to characterize the mechanisms underlying pathological processes, since they lead to negative changes in the central auditory system. These changes create in turn a basis for several hearing disorders by an induction of compensatory, irreversible physiological changes (neuronal reorganization, hyperactivity, hyperexcitability), accompanied by chronic audiological disorders (tinnitus, hyperacusis, reduced ability to process complex sounds, impaired speech processing). Due to the irreversible degeneration, late therapeutic interventions aimed at prevention of noise-induced pathologies are not feasible.

Our present work was designed to clarify the role of specific genes and proteins in the early degeneration of the central auditory system. Based on our recent studies that demonstrated cell loss in the auditory brain as well as on former observations of cochlear pathologies, the experiments started with commercial PCR cell death gene arrays with 84 genes in the cochlear nucleus (CN), inferior colliculus (IC) and auditory cortex (AC) and then particularly focused on the expression of the most frequently regulated anti-apoptotic gene *Bcl2a1a* (B-cell lymphoma 2 related protein a1a) and the related apoptosis-inducing gene *Apaf1* (apoptotic protease activating factor-1). The particular aim was to analyze the possible contribution of mitochondria-related intrinsic apoptotic pathways on acute cellular damage of auditory brain structures immediately after traumatizing noise exposure. We have chosen the time point of 15 min following the 3 h of noise exposure based on our earlier, published studies. In addition to physiological changes (neuronal hyperactivity and increased calcium-related activity), we have determined the changes in cell numbers and the types of cell death pathways in several structures of the central auditory system. Our results implied degenerative process occurring after noise exposure. Our most important finding was that the degeneration was detectable immediately after finishing acoustic trauma, which was shown for the first time in these kinds of experiments (Gröschel et al., 2010, 2011, 2014; Coordes et al., 2012; Fröhlich et al., 2017).

The results of this study should help to fill the gap of knowledge between sound-related hyperexcitation of the auditory pathway, i.e., the induction of hearing loss, and central neurodegeneration.

## Materials and methods

### Animals

Eighteen 6 weeks-old female normal hearing mice (NMRI strain) were used in this study. The experimental protocol was approved by the governmental Ethics Commission for Animal Welfare (LaGeSo Berlin, Germany; approval number: G0416/10). This study was carried out in accordance with the recommendations of the EU Directive 2010/63/EU on the protection of animals used for scientific purposes.

### Experimental groups and tissue preparation

In total, nine animals (trauma group) were anesthetized (60 mg/kg ketamine and 6 mg/kg xylazine) and noise-exposed in a soundproof chamber (0.8 × 0.8 × 0.8 m, minimal attenuation 60 dB) for 3 h to broadband white noise (5–20 kHz) at 115 dB sound pressure level (SPL). The selected noise trauma paradigm is in accordance with what is known about the hearing range of mice (≥1 and ≤100 kHz) and people (≥20 Hz and ≤20 kHz), covering the mouse low-frequency hearing range, and is based on our earlier studies on cellular degeneration and detection of cell death mechanisms after noise exposure to ensure similar experimental conditions (Heffner and Heffner, 2007; Gröschel et al., 2010; Reynolds et al., 2010; Coordes et al., 2012; Fröhlich et al., 2017). Noise was delivered binaurally by speakers (HTC 11.19; Visaton, Haan, Germany) placed above the animal's head. The speakers were connected to an amplifier (Tangent AMP-50; Aulum, Denmark) and a DVD player. SPL was calibrated by using a sound level meter (Voltcraft 329; Conrad Electronic, Hirschau, Germany) placed near the animal's ear. The efficacy of anesthesia was controlled by using a video camera placed inside the lighted chamber. Body temperature was maintained at 37°C with a heating pad (Thermolux CM 15 W; Acculux, Murrhardt, Germany) placed under the animal. Another nine animals served as a normal hearing control group. The control animals were treated equally, that is they were anesthetized and kept in the chamber on a heating pad, but were not exposed to noise (sham exposed group).

Mice were investigated within 15 min after the noise/sham exposure. First, they were euthanized by a lethal dose of anesthetics and next they were decapitated. Brains were removed carefully from the skull, rinsed with PBS and transferred immediately into Allprotect Tissue Reagent (Qiagen, Hilden, Germany cat # 76405) at room temperature for further preparation. Tissue samples from cochlear nucleus (CN, dorsal and ventral subdivision), inferior colliculus (IC) and auditory cortex (AC) from both hemispheres were harvested. Based on our long-standing experience in anatomy and histology of the mouse central auditory system, the boundaries of the investigated structures have been recognized by their individual shape and were gently dissected under visual microscopic control and counterchecked in accordance to a mouse brain atlas (Paxinos and Franklin, 2001). For each of the different structures, tissues harvested from three animals of each group were pooled together and stored at −20°C until RNA and protein isolation. Three separate sets of experiments were performed and each included three noise-exposed animals and three controls, respectively. The mRNA isolated during the first and second experiment was used for qRT-PCR. Target protein levels were estimated using tissues from all three sets of experiments. During all experiments, noise trauma was initiated at the same time in the morning to avoid different noise impact due to diurnal variation in noise susceptibility (Meltser et al., 2014).

### RNA and protein isolation, reverse transcriptase reaction

The tissues stored at −20°C were slowly brought to the room temperature (RT) and the Allprotect reagent was removed using Eppendorf pipette with RNAse-free tip–a total volume of 50 μl was left in each tube. Next, 600 μl of RLT buffer (part of the RNeasy Mini Kit, Qiagen, cat. # 74106) was added to the samples followed by homogenization, vortexing, pulse centrifugation and transfer into QIAshredder Homogenizers (Qiagen, cat. # 79656). Following 2 min of centrifugation at 14,000 rpm, the samples were left at RT for 10 min. The mRNA was precipitated with 650 μl of 70% ethanol and the precipitate was brought to the bottom of the tube by 20 s centrifugation at 8,000 rpm. The rest of the RNA isolation was performed strictly according to the manufacturer's instructions.

The eluate, which contained protein, was collected into fresh tube and stored at −80°C until protein purification procedure. When needed, the samples were mixed with 3 ml of ice-cold acetone and incubated at −20°C for 45 min followed by centrifugation for 20 min at 10.000 rpm. The supernatant was aspirated with Eppendorf pipette and the pellet was dried at RT for 20 min. After this, the pellet was resuspended in 40 μl RIPA buffer containing 5% SDS and dissolved in thermal shaker at 95°C and 500 rpm for 8 min. Next, 80 μl of RIPA buffer was added and the samples were centrifuged in a pre-warmed Eppendorf centrifuge at 24°C, 14.000 rpm for 10 min. The supernatant was collected into fresh tube and the protein concentration was determined with Micro BCA Protein Assay Kit, (ThermoScientific, Darmstadt, Germany cat. # 23235).

The reverse transcription reaction was performed with the RT^2^ First Strand Kit, (Qiagen cat. # 330401) according strictly to the manufacturer's instructions. The RNA concentration was measured using NanoDrop and 200 ng of total RNA was used to synthesize cDNA. Thermal cycler 2720 (Applied Biosystems) was used for sample incubation.

### RT^2^-PCR array experiments

The noise-induced expression of 84 cell death related genes have been profiled using a commercial PCR array in the first part of experiments. The cDNA templates were mixed with ready-to-use SYBR Green Mastermix (RT^2^ SYBR Green qPSR Mastermix, Qiagen, Cat. No. 330502), and loaded onto the 96-well RT^2^-PCR array (PAMM-212ZF-6- RT^2^ Profiler™ PCR Array Mouse Cell Death Pathway Finder, Qiagen, Cat. No. 330231; see Table 1 for detailed list of genes). In the first set of experiments, 49.50 ng of cDNA per well was used for the AC, 61.39 ng of cDNA per well was used for the IC and 29.70 ng of cDNA per well was used for the CN. In the second set of experiments the amounts of cDNA used were different and consisted of 56.44 ng of cDNA per well for the AC, 46.53 ng of cDNA per well for the IC and 36.63 ng of cDNA per well for the CN. Real-time PCR detection was initiated by heating at 95°C for 10 min, followed by 45 cycles of 95°C for 15 s and 60°C for 1 min. The cycling was performed in LightCycler® 96 Instrument (Roche, Mannheim, Germany) and the threshold value Ct was determined by LightCycler® 96 Application and Instrument Software (Roche).

**Table 1:** Gene table RT^2^ PCR array.

**Position**	**UniGene**	**GenBank**	**Symbol**	**Description**
A01	Mm.318925	NM_145557	9430015G10 Rik	RIKEN cDNA 9430015G10 gene
A02	Mm.1318	NM_009594	Abl1	C-abl oncogene 1, non-receptor tyrosine kinase
A03	Mm.6645	NM_009652	Akt1	Thymoma viral proto-oncogene 1
A04	Mm.220289	NM_009684	Apaf1	Apoptotic peptidase activating factor 1
A05	Mm.277585	NM_007471	App	Amyloid beta (A4) precursor protein
A06	Mm.9852	NM_026217	Atg12	Autophagy-related 12 (yeast)
A07	Mm.272972	NM_029846	Atg16l1	Autophagy-related 16-like 1 (yeast)
A08	Mm.41775	NM_026402	Atg3	Autophagy-related 3 (yeast)
A09	Mm.22264	NM_053069	Atg5	Autophagy-related 5 (yeast)
A10	Mm.275332	NM_028835	Atg7	Autophagy-related 7 (yeast)
A11	Mm.396107	NM_023179	Atp6v1g2	ATPase, H+ transporting, lysosomal V1 subunit G2
A12	Mm.19904	NM_007527	Bax	Bcl2-associated × protein
B01	Mm.257460	NM_009741	Bcl2	B-cell leukemia/lymphoma 2
B02	Mm.425593	NM_009742	Bcl2a1a	B-cell leukemia/lymphoma 2 related protein A1a
B03	Mm.238213	NM_009743	Bcl2l1	Bcl2-like 1
B04	Mm.141083	NM_009754	Bcl2l11	BCL2-like 11 (apoptosis facilitator)
B05	Mm.178947	NM_019584	Becn1	Beclin 1, autophagy related
B06	Mm.335659	NM_007465	Birc2	Baculoviral IAP repeat-containing 2
B07	Mm.2026	NM_007464	Birc3	Baculoviral IAP repeat-containing 3
B08	Mm.210125	NM_138313	Bmf	Bcl2 modifying factor
B09	Mm.1051	NM_009807	Casp1	Caspase 1
B10	Mm.3921	NM_007610	Casp2	Caspase 2
B11	Mm.34405	NM_009810	Casp3	Caspase 3
B12	Mm.281379	NM_009811	Casp6	Caspase 6
C01	Mm.35687	NM_007611	Casp7	Caspase 7
C02	Mm.88829	NM_015733	Casp9	Caspase 9
C03	Mm.67659	NM_028492	Ccdc103	Coiled-coil domain containing 103
C04	Mm.271833	NM_011611	Cd40	CD40 antigen
C05	Mm.4861	NM_011616	Cd40lg	CD40 ligand
C06	Mm.336848	NM_009805	Cflar	CASP8 and FADD-like apoptosis regulator
C07	Mm.41687	NM_025417	Commd4	COMM domain containing 4
C08	Mm.236553	NM_007798	Ctsb	Cathepsin B
C09	Mm.3619	NM_021281	Ctss	Cathepsin S
C10	Mm.24282	NM_173369	Cyld	Cylindromatosis (turban tumor syndrome)
C11	Mm.431316	NM_007843	Defb1	Defensin beta 1
C12	Mm.222473	NM_001162917	Dennd4a	DENN/MADD domain containing 4A
D01	Mm.41433	NM_010044	Dffa	DNA fragmentation factor, alpha subunit
D02	Mm.250414	NM_011993	Dpysl4	Dihydropyrimidinase-like 4
D03	Mm.260943	NM_198303	Eif5b	Eukaryotic translation initiation factor 5B
D04	Mm.9213	NM_007956	Esr1	Estrogen receptor 1 (alpha)
D05	Mm.1626	NM_007987	Fas	Fas (TNF receptor superfamily member 6)
D06	Mm.3355	NM_010177	Fasl	Fas ligand (TNF superfamily, member 6)
D07	Mm.32926	NM_023907	Foxi1	Forkhead box I1
D08	Mm.4793	NM_008064	Gaa	Glucosidase, alpha, acid
D09	Mm.72235	NM_007836	Gadd45a	Growth arrest and DNA-damage-inducible 45 alpha
D10	Mm.484118	NM_172855	Galnt5	UDP-N-acetyl-alpha-D-galactosamine:polypeptide N-acetylgalactosaminyltransferase 5
D11	Mm.439649	NM_008163	Grb2	Growth factor receptor bound protein 2
D12	Mm.45272	NM_175111	Hspbap1	Hspb associated protein 1
E01	Mm.209071	NM_010414	Htt	Huntingtin
E02	Mm.240327	NM_008337	Ifng	Interferon gamma
E03	Mm.268521	NM_010512	Igf1	Insulin-like growth factor 1
E04	Mm.275742	NM_010513	Igf1r	Insulin-like growth factor I receptor
E05	Mm.4946	NM_008387	Ins2	Insulin II
E06	Mm.29938	NM_008326	Irgm1	Immunity-related GTPase family M member 1
E07	Mm.306870	NM_020605	Jph3	Junctophilin 3
E08	Mm.252514	NM_027398	Kcnip1	Kv channel-interacting protein 1
E09	Mm.241355	NM_010758	Mag	Myelin-associated glycoprotein
E10	Mm.196239	NM_025735	Map1lc3a	Microtubule-associated protein 1 light chain 3 alpha
E11	Mm.21495	NM_016700	Mapk8	Mitogen-activated protein kinase 8
E12	Mm.1639	NM_008562	Mcl1	Myeloid cell leukemia sequence 1
F01	Mm.256765	NM_008689	Nfkb1	Nuclear factor of kappa light polypeptide gene enhancer in B-cells 1, p105
F02	Mm.475715	NM_030152	Nol3	Nucleolar protein 3 (apoptosis repressor with CARD domain)
F03	Mm.377733	NM_146881	Olfr1404	Olfactory receptor 1404
F04	Mm.277779	NM_007415	Parp1	Poly (ADP-ribose) polymerase family, member 1
F05	Mm.281482	NM_009632	Parp2	Poly (ADP-ribose) polymerase family, member 2
F06	Mm.194127	NM_181414	Pik3c3	Phosphoinositide-3-kinase, class 3
F07	Mm.227506	NM_027514	Pvr	Poliovirus receptor
F08	Mm.26994	NM_016899	Rab25	RAB25, member RAS oncogene family
F09	Mm.394280	NM_028259	Rps6kb1	Ribosomal protein S6 kinase, polypeptide 1
F10	Mm.291525	NM_199422	S100a7a	S100 calcium binding protein A7A
F11	Mm.17484	NM_009221	Snca	Synuclein, alpha
F12	Mm.34342	NM_170756	Spata2	Spermatogenesis associated 2
G01	Mm.40828	NM_011018	Sqstm1	Sequestosome 1
G02	Mm.70781	NM_177191	Sycp2	Synaptonemal complex protein 2
G03	Mm.99793	NM_025382	Tmem57	Transmembrane protein 57
G04	Mm.1293	NM_013693	Tnf	Tumor necrosis factor
G05	Mm.193430	NM_020275	Tnfrsf10b	Tumor necrosis factor receptor superfamily, member 10b
G06	Mm.15383	NM_008764	Tnfrsf11b	Tumor necrosis factor receptor superfamily, member 11b (osteoprotegerin)
G07	Mm.1258	NM_011609	Tnfrsf1a	Tumor necrosis factor receptor superfamily, member 1a
G08	Mm.3399	NM_009422	Traf2	Tnf receptor-associated factor 2
G09	Mm.222	NM_011640	Trp53	Transformation related protein 53
G10	Mm.37667	NM_175646	Txnl4b	Thioredoxin-like 4B
G11	Mm.271898	NM_009469	Ulk1	Unc-51 like kinase 1 (C. elegans)
G12	Mm.259879	NM_009688	Xiap	X-linked inhibitor of apoptosis
H01	Mm.328431	NM_007393	Actb	Actin, beta (housekeeping gene 1)
H02	Mm.163	NM_009735	B2m	Beta-2 microglobulin (housekeeping gene 2)
H03	Mm.343110	NM_008084	Gapdh	Glyceraldehyde-3-phosphate dehydrogenase (housekeeping gene 3)
H04	Mm.3317	NM_010368	Gusb	Glucuronidase, beta (housekeeping gene 4)
H05	Mm.2180	NM_008302	Hsp90ab1	Heat shock protein 90 alpha (cytosolic), class B member 1 (housekeeping gene 5)
H06	N/A	SA_00106	MGDC	Mouse Genomic DNA Contamination Control
H07	N/A	SA_00104	RTC	Reverse Transcription Control
H08	N/A	SA_00104	RTC	Reverse Transcription Control
H09	N/A	SA_00104	RTC	Reverse Transcription Control
H10	N/A	SA_00103	PPC	Positive PCR Control
H11	N/A	SA_00103	PPC	Positive PCR Control
H12	N/A	SA_00103	PPC	Positive PCR Control

Changes in mRNA levels were expressed as fold increase/decrease. The cut-off determining expression was ≥2.0 or ≤–2.0 fold changes. Genes, which met these criteria, were considered to be upregulated or downregulated.

Of the 84 cell death related genes monitored for mRNA expression in this part of the experiments, *Bcl2a1a* and *Apaf1* were selected for further detailed quantitative analysis of noise-induced alterations in mRNA and protein levels. During all experiments, expression of three housekeeping genes present on each of the arrays was used for normalization: beta-actin, beta-2 microglobulin and heat shock protein 90 alpha (cytosolic), class B member 1.

### RT^2^-qPCR primer experiments

The sets of forward and reverse primers specific for *Apaf1* and *Bcl2a1a* were purchased from Qiagen. These primers sets were identical to the ones spotted on the expression arrays and their sequence is protected by patent. We used the primers pairs to perform confirmatory RT-PCR using this time not the pooled but individual cDNA samples. The cycling conditions were identical to the conditions used for the arrays.

### Protein analyses

The protein concentration was measured and samples containing were mixed with Roti-Load 1 (Carl Roth, Karlsruhe, Germany, cat. #K929.1). Samples containing 16 μg of total protein were pipetted into the wells of SDS-PAGE gel (Novex WedgeWell 4–20% Tris-Glycine Gel, 12 well, Thermo Fisher Scientific, cat. # XPO4202BOX). PageRuler Prestained Protein Ladder was used to estimate the molecular weight (Thermo Scientific cat. # 26618). Following the electrophoresis in Xcell SureLock Electrophoresis Cell, Invitrogen, the proteins were blotted onto Immobilon-P Transfer Membrane 0.45 μm, (Millipore cat. # IPFL 000 10) with use of XCell II Blot Module (Invitrogen cat. # EI9051). The membrane was blocked in 5% skimmed milk/PBS/0.05%Tween buffer for 1 h after which, incubation with primary and secondary antibodies followed. To detect 39 kDa BCL2A1A, a rabbit polyclonal antibody was used (Abcam # ab45414) in dilution of 1:5000. To detect 130 kDa APAF1, rabbit polyclonal antibody (Enzo # ADI-905-179) was used in dilution of 1:500. The blots were incubated with primary antibodies overnight at 4°C, followed by triple wash in PBS/0.1%Tween. Secondary antibody used was in both cases goat-anti-Rabbit IgG (H+L), conjugated with horseradish peroxidase (Promega, cat. # W4011) at the dilution of 1:5000. Protein was visualized by adding Pierce ECL Western Blotting Substrate (Thermo Scientific cat. # 32209) and scanning by C-DiGit Blot Scanner, LI-COR (Bad Homburg, Germany), followed by image analyses with Image Studio Version 5x. The unique technology of C-Digit allows direct measurement of chemiluminescence induced by a presence of enzyme-labeled secondary antibody bound to the target protein blotted on a membrane and therefore, allows a reduction of one experimental step and improves accuracy of the result.

### Statistical analyses

The data obtained from the noise-exposure animals were compared to data obtained from the control group in each investigated brain region. Delta Cq values from the expression arrays and calculated ratios to controls obtained from the western blot were used for statistical comparison between BCL2A1A and APAF1 groups. Depending on data distribution, *U*-test (not normally distributed data) or *t*-test (normally distributed data) was used. Data distribution was tested by Shapiro-Wilk test. The software SPSS (IBM SPSS Statistics Version 23, IBM Corp., Armonk, New York, USA) was used for all statistical analyses. The alpha level of significance was set for <0.05. Data are presented as mean ± standard deviation.

## Results

### RT^2^-PCR array experiments

In the first set of experiments, commercial RT-PCR expression arrays were used to determine changes in the expression of 84 cell death-related genes (Table 1). Upregulation (≥2.0 fold expression changes) of gene expression was observed only for one gene (*Hspbap1*) in the inferior colliculus and for one gene (*Hspbap1*) in the primary auditory cortex, whereas no changes have been detected in the cochlear nucleus. Expressional downregulation (≤–2.0 fold expression changes) affected three genes (*Cd40, Esr1, Galnt5*) in the CN, 11 genes (*Akt1, Apaf1, Bcl2a1a, Casp6, Fas, Galnt5, Igf1, Ins2, Sycp2, Tmem57, Tnf*) in the IC and eight genes (*Bcl2a1a, Bmf, Cd40, Defb1, Fas, Fasl, Foxi1, Olfr1404*) in the AC.

In the second set of experiments using a different amount of cDNA, we detected an upregulation of 12 genes (*Atg5, Bcl2a1a, Bcl2l11, Casp1, Casp6, Ctsb, Dpysl4, Eif5b, Igf1, Ins2, Mag, Rab25*) in the CN, 13 genes (*Akt1, Atg7, Atp6v1g2, Bcl2l11, Casp1, Foxi1, Hspbap1, Mag, Parp1, S100a7a, Snca, Tmem57, Tnfrsf1a*) in the IC and for 15 genes (*Bcl2a1a, Bcl2l1, Birc2, Bmf, Cd40, Cd40lg, Defb1, Fas, Fasl, Gaa, Galnt5, Ifng, Kcnip1, Mag, Olfr1404*) in the AC. Downregulation was present for 2 genes (*Apaf1, Defb1*) in the CN, for 17 genes (*Abl1, Bcl2a1a, Bmf, Casp6, Cd40, Cd40lg, Cflar, Defb1, Dffa, Fas, Gadd45a, Galnt5, Ifng, Igf1, Ins2, Olfr1404, Rab25*) in the IC and 6 genes (*Casp1, Ccdc103, Foxi1, Ins2, S100a7a, Snca*) in the auditory cortex (see Table 1 for list of abbreviations).

For detailed documentation, data of gene expression analysis from both sets of experiments is available in the Supplementary Material (Supplementary Tables 1–6).

### Confirmatory RT-PCR experiments

Gene expression analysis demonstrated significant changes in the expression of both investigated genes, *Bcl2a1a* and *Apaf1*, following an acute acoustic overstimulation (trauma group, 6 animals, number of samples: *n* = 8) compared to an unexposed control group (6 animals, number of samples: *n* = 8). These particular genes were selected as the anti-apoptotic gene *Bcl2a1a* was the most frequently regulated gene in both sets of RT^2^-PCR array experiments and the pro-apoptotic *Apaf1* gene represents one of the central stages in the related apoptosis regulatory network.

**Figure 1:**
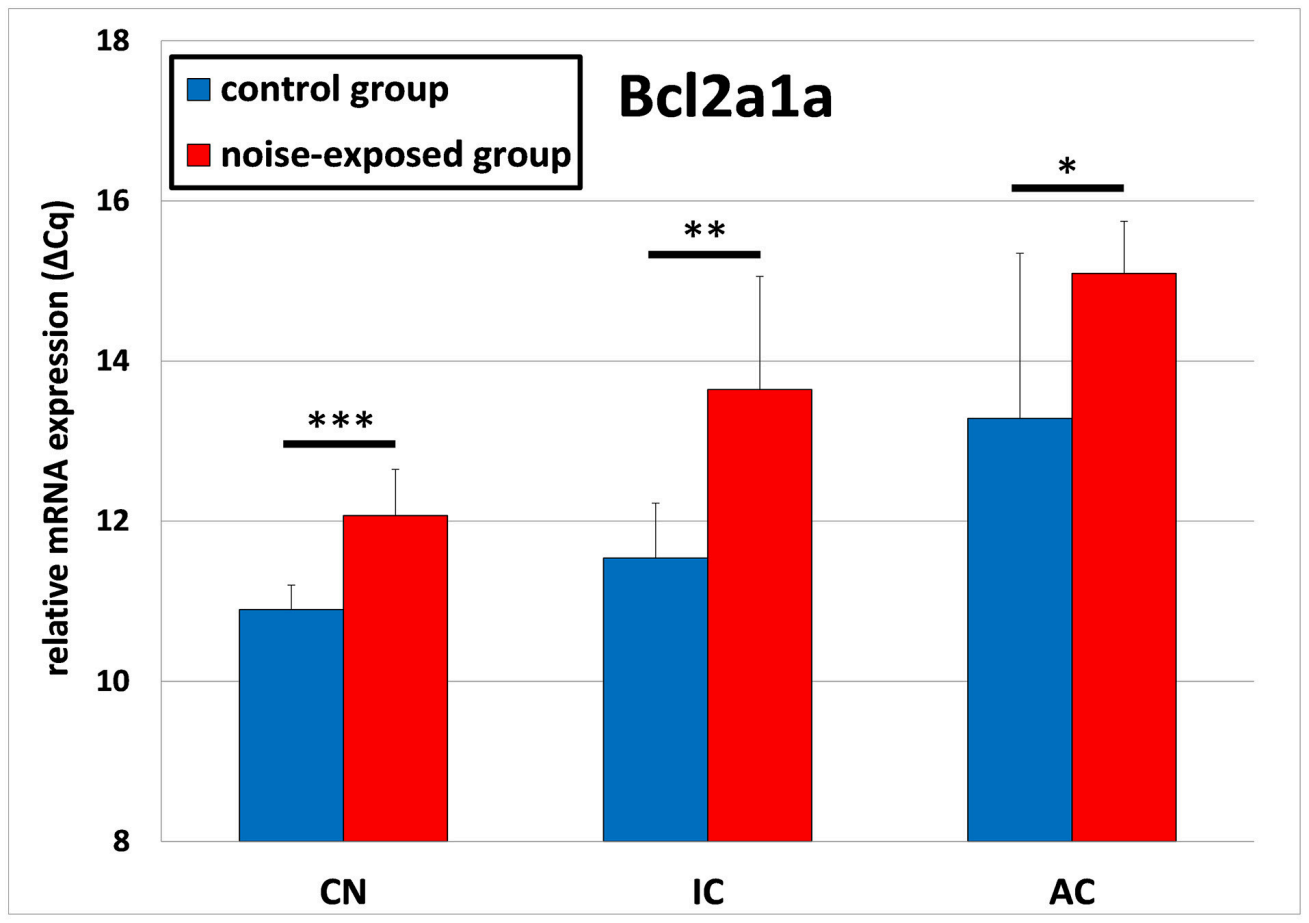
Relative mRNA expression of *Bcl2a1a*. Presented are ΔCq values (mean ± S.D.) in the cochlear nucleus (CN), inferior colliculus (IC) and auditory cortex (AC) of the mouse central auditory pathway immediately after noise trauma compared to unexposed controls. Asterisks indicate significant differences between control and trauma group (*p < 0.05; **p < 0.01; ***p < 0.001).

The expression of *Bcl2a1a* was upregulated in all investigated structures of the central auditory pathway (Figure 1). In the cochlear nucleus, mean ΔCq value was significantly greater in the trauma group 12.089 ± 0.578 than in control animals 10.895 ± 0.307 (*p* < 0.001; *t*-test; *t* = −5.073; *df* = 14; effect size d: 2.580; power: 0.999). Similar *Bcl2a1a* expression pattern could be seen in the inferior colliculus as well as in the auditory cortex. In the IC, mean ΔCq values were 11.539 ± 0.687 in the controls and 13.644 ± 1.413 in the noise-exposed animals [*p* = 0.002; *t*-test (*t* = −3.789; *df* = 14; effect size d: 1.895; power: 0.975)]. In the auditory cortex, ΔCq values rose from a mean ΔCq of 13.283 ± 2.063 in the control group to 15.095± 0.650 in the noise-exposed group [*p* = 0.044; *t*-test (*t* = −2.370; *df* = 14; effect size d: 1.185; power: 0.728)].

**Figure 2:**
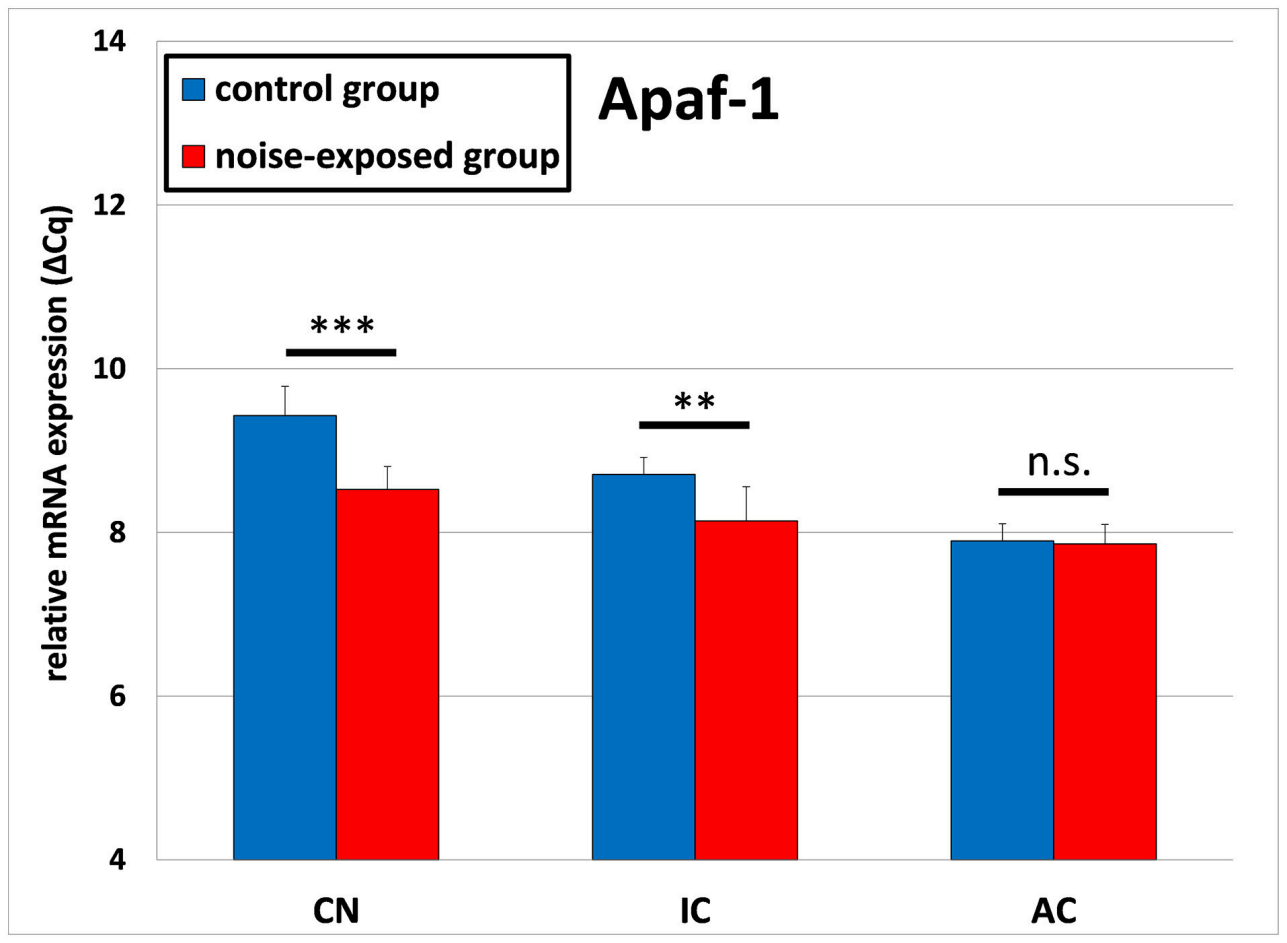
Relative mRNA expression of *Apaf1*. Presented are ΔCq values (mean ± S.D.) in the cochlear nucleus (CN), inferior colliculus (IC) and auditory cortex (AC) of the mouse central auditory pathway immediately after noise trauma compared to unexposed controls. Asterisks indicate significant differences between control and trauma group (**p < 0.01; ***p < 0.001; n.s., no significant difference between groups).

The gene expression analysis of *Apaf1* delivered opposite results (Figure 2). The ΔCq values in the noise-exposed group were significantly lower as these in the control group. In detail, cochlear nucleus of the control group had the mean ΔCq of 9.428 ± 0.359 whereas cochlear nucleus of the noise-exposed animals had the mean ΔCq of 8.525 ± 0.282 [*p* < 0.001; *t*-test (*t* = 5.595; *df* = 14; effect size d: 2.797; power: 1)]. Similarly, the mean ΔCq in inferior colliculus of the control group was 8.710 ± 0.207 whereas in the noise-exposed group ΔCq was 8.141 ± 0.418 [*p* = 0.004; *t*-test (*t* = 3.450; *df* = 14; effect size d: 1.725; power: 0.947)]. No significant differences in the expression of *Apaf1* gene have been observed between both groups in the auditory cortex [control group: 7.896 ± 0.211, trauma group: 7.860 ± 0.238; *p* = 0.752; *t*-test (*t* = 0.322; *df* = 14)].

### Western blot experiments

The relative protein levels of BCL2A1A and APAF1 were compared between the noise-exposed group (9 animals, number of samples: *n* = 12) and the control group (9 animals, number of samples: *n* = 12) for each investigated structure separately (see Supplementary Figure 1 in Supplementary Material for original data from the western blot experiments).

**Figure 3:**
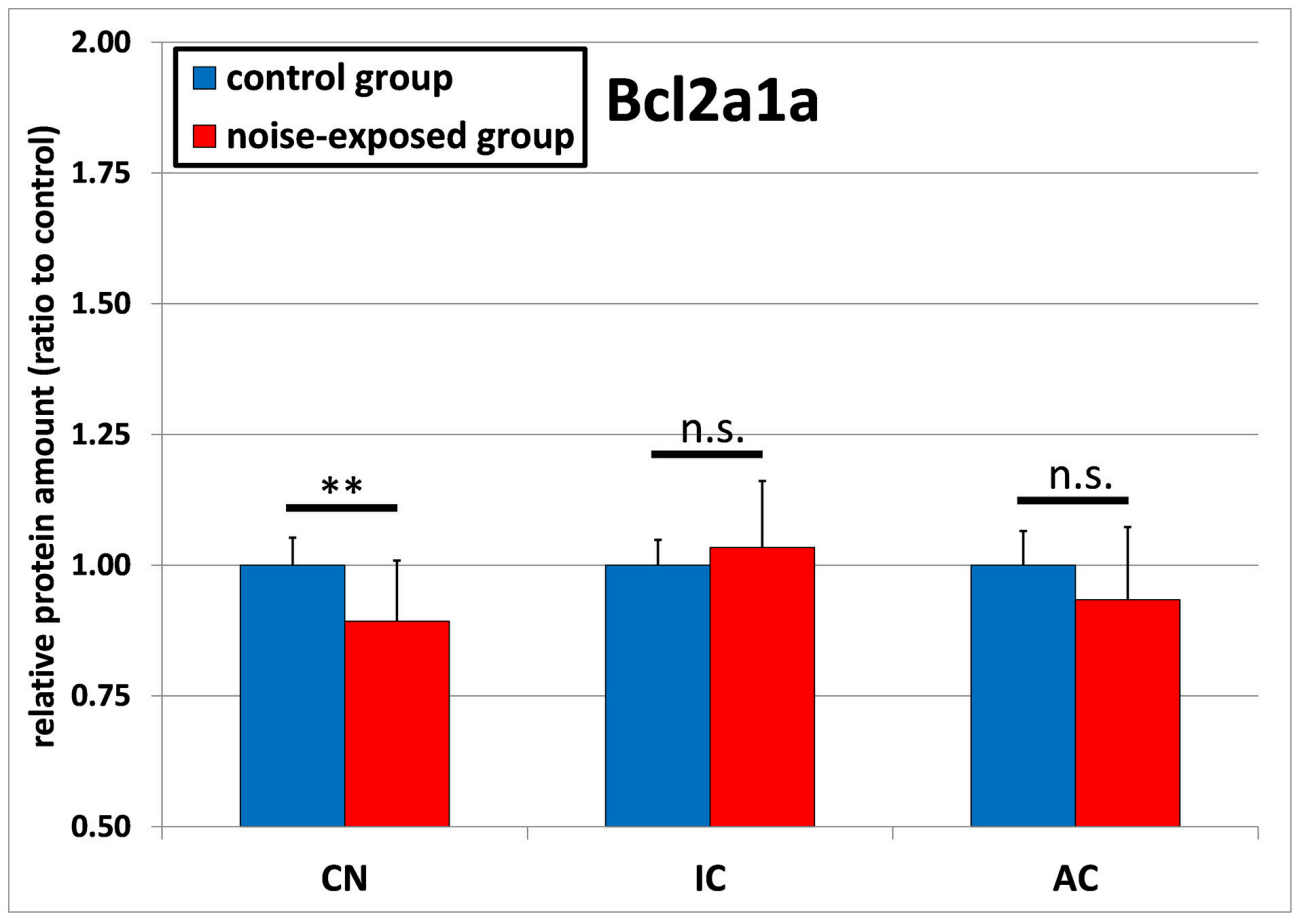
Relative protein amounts of BCL2A1A. Presented are ratios to respective controls from the Western blot experiments (mean ± S.D.) in the cochlear nucleus (CN), inferior colliculus (IC) and auditory cortex (AC) of the mouse central auditory pathway immediately after noise trauma compared to unexposed controls. Asterisks indicate significant differences between control and trauma group (**p < 0.01; n.s., no significant difference between groups).

BCL2A1A protein levels were shown to be significantly decreased in response to acoustic trauma in the cochlear nucleus. No significant changes could be detected in the inferior colliculus or auditory cortex (Figure 3). In the CN, relative mean ratio of experimental group to control (1.0 ± 0.053) was 0.893 ± 0.116, which was significantly less than the control [*p* = 0.008; *t*-test (*t* = 2.901; *df* = 22; effect size d: 1.187; power: 0.942)]. For the inferior colliculus, there were no statistically significant differences between groups [control group: 1.0 ± 0.049, noise-exposed group: 1.034 ± 0.127; *p* = 0.908; *U*-test (*U* = 70; *df* = 22)]. The same holds true for the auditory cortex, showing a slight but not significant reduction in protein amount ratio between groups [control group: 1.0 ± 0.065, noise-exposed group: 0.934 ± 0.139; *p* = 0.147; *t*-test (*t* = 1.502; *df* = 22)].

**Figure 4:**
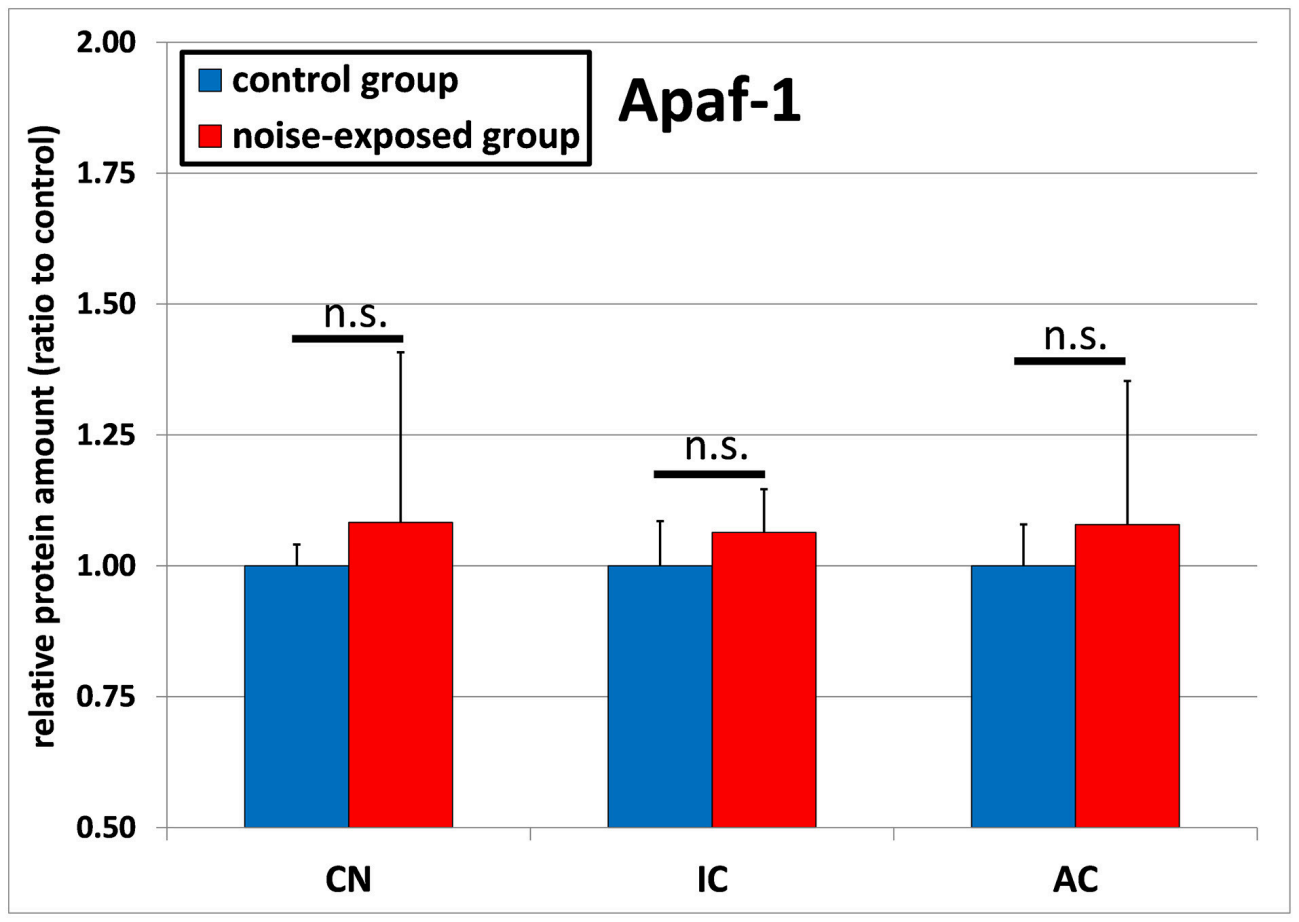
Relative protein amounts of APAF1. Presented are ratios to the respective controls from the Western blot experiments (mean ± S.D.) in the cochlear nucleus (CN), inferior colliculus (IC) and auditory cortex (AC) of the mouse central auditory pathway immediately after noise trauma compared to unexposed controls (n.s., no significant difference between groups).

The protein levels of APAF1 were slightly greater in all investigated structures of the ascending central auditory pathway, although missing statistical significance (Figure 4). In the inferior colliculus, statistical comparison of mean expression ratio between noise-exposed animals and unexposed controls showed *p*-value of 0.074 [control group: 1.0 ± 0.085, trauma group: 1.064 ± 0.082; *t*-test (*t* = −1.874; *df* = 22)]. In addition, this difference was also not statistically significant for cochlear nucleus [control group: 1.0 ± 0.041, noise-exposed group: 1.083 ± 0.325; *p* = 0.396; *t*-test (*t* = −0.882; *df* = 22)] or for the auditory cortex [control group: 1.0 ± 0.079, noise-exposed group: 1.079 ± 0.247; *p* = 0.773; *U*-test (*U* = 67; *df* = 22)].

## Discussion

The present study investigates acute noise-induced changes in the expression of genes encoding members of the intrinsic apoptotic pathway, *Bcl2a1a* and *Apaf1* in the key structures of the auditory pathway: cochlear nucleus, inferior colliculus and auditory cortex. Moreover, we investigated changes in the protein levels of BCL2A1A and APAF1 in the same structures. As it was the aim of this study to determine gene and protein expression immediately after noise exposure, therefore the auditory function in these particular animals could not be tested. However, in our earlier studies using the same noise exposure protocol (covering the mouse low-frequency hearing range) in NMRI mice, we observed greatest hearing loss immediately after noise exposure (mean threshold shift between 4 and 20 kHz: ≥60 dB). The hearing thresholds recovered partially over 1 week, and the animals were left with a stable mean permanent threshold shift of 38 dB between 4 and 20 kHz (Gröschel et al., 2011). Moreover, several other earlier studies reported that identical treatment caused the development of spontaneous neuronal hyperactivity (possibly related to tinnitus) as well as an acute and long-term upregulation in calcium activity and cellular degeneration in auditory brain structures (Gröschel et al., 2010, 2011, 2014; Coordes et al., 2012; Fröhlich et al., 2017). Our results demonstrated upregulation of *Bcl2a1a* gene expression immediately after acoustic trauma in all investigated structures, whereas the protein levels were reduced at least in the auditory brainstem. On the other hand, the transcriptional expression of *Apaf1* mRNA was reduced after acoustic overexposure along the auditory pathway. The protein levels of APAF1 tend to be increased but without statistical significance.

### Degeneration in the auditory system

Apoptosis, the process of programmed cell death, occurs normally during development, aging or as a homeostatic mechanism and is essential for multicellular organisms. However, despite other cell death pathways, apoptosis might also occur in pathological conditions and is a key process contributing to neurodegenerative diseases, ischemia and autoimmune disorders (Elmore, 2007). Hearing disorders occurring due to acoustic trauma, pharmacological exposure or due to direct damage in the auditory periphery have the ability to induce apoptosis not only in the cochlear structures but also in the auditory nerve and the central auditory system (Coordes et al., 2012; Yu et al., 2014; Ding et al., 2015; Bohne et al., 2017). Although acute and long-term necrosis has been detected and the expression of necrosis-related genes changes, apoptosis seems to be a key mediator of cell death in NIHL particularly induced by high frequency noise at high sound intensities (Hu et al., 2000, 2002; Coordes et al., 2012; Yu et al., 2014; Bohne et al., 2017). Studies of the peripheral and central auditory system have shown the impact of noise on auditory tissues already within 1 h after exposure, being progressive for weeks and months. The degenerative effects are most prominent in cochlear outer hair cells followed by inner hair cells, supporting cells and further delayed negative effect along the central auditory pathway (Hamernik et al., 1984; Yang et al., 2004; Gröschel et al., 2010; Meltser et al., 2010; Coordes et al., 2012; Bohne et al., 2017; Fröhlich et al., 2017). Several studies investigated the expression of different cell death-related genes during cochlear degeneration to give insight into the underlying mechanisms.

### Early pathologies in the cochlea

Apoptotic mechanisms have been characterized during exposure of cochlea to pharmacological agents *in vitro* and *in vivo*. Cisplatin-treated cochlear explants express several pro- and anti-apoptotic genes, particularly *p53* and the members of *Bcl2* family. In addition, caspase activation and an increase in stress response proteins within a few hours after treatment has been demonstrated (Devarajan et al., 2002; Coling et al., 2007; Ding et al., 2009; Wang et al., 2013). *In vivo* experiments with ototoxic drugs indicated a large contribution of genes and proteins related to intrinsic apoptosis signaling pathway (García-Berrocal et al., 2007). Moreover, an early increase in oxidative stress responses, contributing to particular cell death pathways, and accompanied by apoptotic and inflammatory reactions on the gene level in cochlear tissue, has been described in cochlea (Kirkegaard et al., 2006; Mazurek et al., 2011; Gross et al., 2014).

Also noise exposure has been shown to induce early changes in the expression of several pro- and anti-apoptotic genes in cochlea (Han et al., 2011). Noise exposure leads to neuronal hyperexcitation, which is followed by ischemia-induced generation of ROS and initiation of apoptosis in the auditory system. Oxidative stress that follows and plays an important role during intrinsic apoptosis, is a key mechanism responsible for acquired hearing loss (Henderson et al., 2006). On the cochlear level, an acute upregulation of pro-apoptotic TNF family members and a downregulation of anti-apoptotic *Bcl2*-related genes was demonstrated 4 h after noise exposure (Hu et al., 2009). On the other hand, an upregulation of anti-apoptotic genes was present within 1 h after sound overexposure (Han et al., 2012).

It is a common feature of a large number of early regulated genes in evoked hearing loss that they are involved in the intrinsic apoptotic pathway. A detailed examination of caspase activity in the peripheral auditory system supports the idea about the intrinsic apoptotic pathway being particularly responsible for the ototoxic and age-related cell death (upregulation of caspases 3 and 9, participation of pro-apoptotic BAK-dependent pathway), while both, the extrinsic and intrinsic pathway, contribute to the noise-induced hearing loss (activation of caspases 3, 8, and 9) (Hu et al., 2002; Nicotera et al., 2003; Van De Water et al., 2004).

Comparable changes of intrinsic pro- and anti-apoptotic signaling, including regulation of BCL2 family members and APAF1, were also shown during age-related hearing loss (ARHL) (Alam et al., 2001; Nevado et al., 2006; Tadros et al., 2008; Falah et al., 2016).

### Intrinsic apoptosis and the central auditory system

While the extrinsic apoptotic pathway is induced by transmembrane receptor interactions involving death ligands and corresponding death receptor domains of the tumor necrosis factor family (Ashkenazi and Dixit, 1998), the intrinsic apoptotic pathway is induced by a non-receptor-mediated stimuli, acting directly on intracellular targets and being initiated in the mitochondria (Elmore, 2007). Activation of intrinsic apoptosis occurs through changes at the inner mitochondrial membrane, which alters the transmembrane potential. The release of cytochrome c from mitochondria into the cytosol activates APAF1 to facilitate the formation of apoptosome, which in turn triggers proteolytic caspase cascades (pro-caspase 9, caspase 9 and “executioner” caspase 3) and leads to apoptosis (Sakahira et al., 1998; Hill et al., 2004; Saelens et al., 2004). APAF1 is a key molecule in the intrinsic mitochondrial pathway and conformational changes occur by binding of cytochrome c (Jiang and Wang, 2000; Shakeri et al., 2017). The direct involvement of APAF1 in ototoxicity was shown by diminishing the chemotherapy-related hearing loss trough pharmacological inhibition of APAF1 (Orzáez et al., 2014). The levels of APAF1 protein are generally low and thus, are a limiting factor in apoptosome formation and apoptotic signaling. Therefore, even a slight increase demonstrated in the present study could substantially contribute to apoptosis (Yoshida et al., 1998; Shakeri et al., 2017).

The BCL2 family consists of several pro- and anti-apoptotic genes and related proteins. BCL2 family members, including anti-apoptotic BCL2A1A, alter mitochondrial membrane permeability (Cory and Adams, 2002). Pro-apoptotic BCL2-related proteins (including BAX, BAK and BH3-only proteins) have the ability to induce the formation of pores in the outer mitochondrial membrane and to mediate cytochrome c release, which is the critical step in the intrinsic apoptotic pathway (Vogler, 2012). Imaging experiments have shown that the neuronal calcium uptake is strongly increased immediately after acoustic trauma, particularly in the auditory brainstem (Gröschel et al., 2011). Calcium plays an important role in mitochondrial homeostasis and is a key mediator of neuronal ischemia-induced excitotoxicity. Therefore, neuronal hyperexcitation can trigger intrinsic apoptosis by directly increasing the level of oxidative stress and ROS formation; thus, further influencing mitochondrial membrane potential (Yu et al., 2001; Salinska et al., 2005; Mattson, 2007).

Anti-apoptotic proteins of the BCL2 family generally counteract the activation of pro-apoptotic proteins. In the present study, this effect might be represented by the strong increase of *Bcl2a1a* gene expression. Such upregulated transcriptional expression may prevent further apoptosis by subsequently increasing BCL2A1A protein synthesis. According to the present results, an increase of intrinsic anti-apoptotic Bcl2a1 a signaling was demonstrated in the inferior colliculus 2 days after exposure to cisplatin (Manohar et al., 2014). Upregulation of anti-apoptotic genes, often along with increased stress protein responses, are supposed to represent a cellular mechanism of self-defense in response to pro-apoptotic gene and protein expression following acoustic injury, compensating for activation of cellular damage and balancing pro- and anti-apoptotic machineries (Wada and Penninger, 2004; Coling et al., 2007; Someya et al., 2009; Portt et al., 2011; Wang et al., 2013). This notion is supported by studies showing an acute upregulation of heat shock proteins in response to noise-induced peripheral and central degeneration, further initiating recovery from acutely developing central hyperactivity and promoting cell survival (Helfert et al., 2002; Samson et al., 2007; Sun et al., 2008).

Changes in levels of particular mRNA and proteins are related to each other in a time-dependent manner, meaning that greater amount of a particular protein could induce downregulation of the respective gene transcription (and vice versa) representing a homeostatic mechanism to rebalance the cellular proteome, referred to as proteostasis (Balch et al., 2008; Douglas and Dillin, 2010). The changes in gene and protein expression do not occur simultaneously and there may be substantial time difference between visible increase in gene expression and that of protein expression. The time window, which we studied, was rather narrow and consisted of merely 3 h. In case of possible gene expression activation, the protein synthesis may follow significantly later (Hargrove et al., 1991). For instance, we have shown earlier that the induction of HSP72 gene expression occurs already 2 h after exposure to geldanamycin, whereas statistically different protein synthesis follows 4 h after induction (Yu et al., 2009). In case of gene expression inhibition, then this also not necessarily has to be mirrored by an immediate decrease of protein concentration. In fact, half-life of most of the proteins can be measured in hours whereas that of mRNA – in minutes or even seconds, depending on if and how is mRNA protected (Friedel et al., 2009). This concept is in line with the present results, since in our study, the levels of anti-apoptotic BCL2A1A protein were significantly lower at least in the cochlear nucleus, possibly due to increased intrinsic apoptosis after acoustic trauma. Consistently, the levels of pro-apoptotic APAF1protein were slightly increased in the CN. Although missing the significance level of 5% (*p* = 0.074), which is possibly related to the noise trauma paradigm only covering the mouse low-frequency hearing range, an increased level of intrinsic apoptosis would be able to explain the relatively strong reduction in *Apaf1* as well as the elevated *Bcl2a1a* mRNA expression, indicating a compensating cellular reaction to counteract cellular injury. An acute upregulation of apoptosis within the auditory brainstem is in line with earlier studies, demonstrating activation of pro-apoptotic JNK cascades, increased levels of single-stranded DNA breaks characteristic for apoptosis and an acute reduction in cell number immediately after noise exposure, with the strongest impact in the lower structures and diminishing effects toward the auditory cortex (Gröschel et al., 2010; Meltser et al., 2010; Coordes et al., 2012; Fröhlich et al., 2017).

Downregulation of pro-apoptotic and upregulation of anti-apoptotic genes was also shown in the cochlear nucleus during postnatal development in mice, presumably to prevent mature neurons from stressful insult, thereby pointing at a common underlying physiological response pattern determined during the development of central nervous system, but possibly occurring in pathological situations (Harris et al., 2005).

On the other hand, APAF1 protein is supposed to function as a pro-survival molecule, which absence impairs the cell performance and increases responsiveness to stressful conditions. Thus, a compensatory reduction of *Apaf1* mRNA expression could probably be accompanied by a negative impact on the structural and functional arrangement of mitochondrial homeostasis and might further contribute to the long-term physiological deficits and pathologies after noise exposure (Ferraro et al., 2011; Sancho et al., 2014; De Zio et al., 2015).

Lastly, the dynamics of transcription/translation do not always work in the canonical way. Examples were described, where despite increase of mRNA levels, the protein levels decreased (Liu et al., 2016). In addition, the primary brain tissue of mice contains 35% of non-neuronal cells and the techniques based on tissue lysis will deliver an average signal per weight unit of tissue, but is not cell-specific (Herculano-Houzel, 2014). Furthermore, transcription and translation process in neurons occur in a different manner than in other cell types and the changes in protein level is often not combined in time and space with changes occurring in gene transcription. In fact, many mRNA species are pre-synthesized in the nucleus and instead of being immediately translated, they are transported near the synapses and there, locally translated as needed (Glock et al., 2017).

### Technical aspects

In this work, we performed two sets of experiments and obtained some overlapping and some non-overlapping results, thus, raising a question about reproducibility of our expression array results. However, we expected this kind of variability. The first reason accounting for variability of results is the technical error, multiplied by a number of manipulations (e.g., RNA isolation, the process of pooling of RNA into one sample or the efficiency of cDNA *in vitro* transcription). The last multiplication of possible differences occurs in logarithmic manner during the PCR. The second reason that may account for the differences in results obtained on two separate occasions is using outbred strain of mice and additional, epigenetic differences between individual animals, which could have affected gene expression pattern. The third and last reason is that the biological reaction of animals to noise may vary depending on many factors, and some of these factors may still be unknown.

It still remains unclear if and how far other cell death mechanisms contribute to the observed degeneration. However, previous studies together with the present data lead to the assumption about the intrinsic pathway playing a key role during the immediate response to traumatic insult of the auditory system. In turn, the extrinsic apoptosis and necrosis may be of greater importance at a later time following noise exposure. Comparable observations concerning the timing of apoptotic pathways have been made e.g., after induction of ischemic stroke (Broughton et al., 2009).

Future experiments should clarify the presence and development of different cell death mechanisms following noise trauma. Moreover, induction of apoptotic or necrotic cell death and neurodegeneration could lead to inflammatory responses that have indeed been observed in the cochlear and auditory brain tissues during oxidative stress or after hearing loss (Kirkegaard et al., 2006; Khan et al., 2010; Broughton et al., 2012; Magnus et al., 2012; Tan et al., 2016; Fuentes-Santamaria et al., 2017). Consequently, cellular damage and inflammation might be accompanied by (or even elicit) the neuroplasticity. Subsequently, neuroplasticity may increase excitatory neurotransmission along the auditory pathway, and possibly contribute to psychoacoustic pathologies such as tinnitus (Oh et al., 2007; Mazurek et al., 2012a,b; Manohar et al., 2016).

In conclusion, our experiments gave insight into the underlying mechanisms of early neurodegeneration of the auditory brain induced by acoustic overexposure. Moreover, for the first time we provided direct evidence about key intrinsic apoptosis genes and their products being expressed or present immediately after noise trauma, respectively. Inhibition of the intrinsic apoptotic pathway could be a promising therapeutic approach for the treatment of acute, noise-induced hearing loss. Such treatment could prevent irreversible neuronal damage and might be of particular importance to block the development of profound deficits in complex central auditory processing.

## Author contributions

MG, DB, AE, BM, and AS: designed the study; MG and AS: performed experiments and analyzed the data; DB: assisted with data analysis; MG and AS: wrote the manuscript; MG, DB, AE, BM, and AS: critically reviewed and approved the final manuscript.

### Conflict of interest statement

The authors declare that the research was conducted in the absence of any commercial or financial relationships that could be construed as a potential conflict of interest.
