# Supplementary material for: Acute Noise Exposure Is Associated With Intrinsic Apoptosis in Murine Central Auditory Pathway
Source: Front Neurosci. 2018 May 9;12:312. doi: 10.3389/fnins.2018.00312 (PMC5954103; doi:10.3389/fnins.2018.00312)
Supplement: Supplementary file 1 [file Data_Sheet_1.docx]

***Supplementary Material***

**Acute Noise Exposure Is Associated With Intrinsic Apoptosis in Murine Central Auditory Pathway**

Moritz Gröschel^1,*^, Dietmar Basta^1^, Arne Ernst^1^, Birgit Mazurek^2^, Agnieszka J. Szczepek^3, *^

^1^ Department of Otolaryngology, Unfallkrankenhaus Berlin, Charité Medical School, Berlin, Germany

^2^ Tinnitus Center, Charité-Universitätsmedizin Berlin, corporate member of Freie Universität Berlin, Humboldt-Universität zu Berlin, and Berlin Institute of Health, Berlin, Germany

^3^ Department of Otorhinolaryngology, Head and Neck Surgery, Charité-Universitätsmedizin Berlin, corporate member of Freie Universität Berlin, Humboldt-Universität zu Berlin, and Berlin Institute of Health, Berlin, Germany

* Correspondence:

Dr. Moritz Gröschel

moritz.groeschel@biologie.hu-berlin.de

Dr. Agnieszka J. Szczepek

agnes.szczepek@charite.de

**Supplementary Tables**

**Supplementary Table 1.** PCR array dataset of the cochlear nucleus (experiment 1), representing fold regulation in expression of the corresponding gene in the trauma group (noise exposed) compared to unexposed controls. Upregulated genes (fold regulation ≥ 2.0) are written in red, downregulated genes (fold regulation ≤ -2.0) are written in blue.

| **Position** | **Gene symbol** | **Fold regulation** |
| --- | --- | --- |
| A01 | 9430015G10Rik | -1.1095 |
| A02 | Abl1 | -1.2226 |
| A03 | Akt1 | -1.2311 |
| A04 | Apaf1 | 1.4743 |
| A05 | App | -1.1567 |
| A06 | Atg12 | 1.2142 |
| A07 | Atg16l1 | 1.1173 |
| A08 | Atg3 | -1.5368 |
| A09 | Atg5 | -1.0069 |
| A10 | Atg7 | -1.0281 |
| A11 | Atp6v1g2 | -1.6244 |
| A12 | Bax | -1.0943 |
| B01 | Bcl2 | 1.6133 |
| B02 | Bcl2a1a | -1.6132 |
| B03 | Bcl2l1 | -1.2311 |
| B04 | Bcl2l11 | -1.3014 |
| B05 | Becn1 | -1.2058 |
| B06 | Birc2 | -1.2311 |
| B07 | Birc3 | 1.2058 |
| B08 | Bmf | -1.7655 |
| B09 | Casp1 | 1.2397 |
| B10 | Casp2 | 1.6702 |
| B11 | Casp3 | -1.1329 |
| B12 | Casp6 | -1.1408 |
| C01 | Casp7 | -1.2571 |
| C02 | Casp9 | -1.9186 |
| C03 | Ccdc103 | -1.8921 |
| C04 | Cd40 | -3.4578 |
| C05 | Cd40lg | -1.1329 |
| C06 | Cflar | 1.1408 |
| C07 | Commd4 | -1.3755 |
| C08 | Ctsb | -1.0943 |
| C09 | Ctss | -1.2142 |
| C10 | Cyld | -1.2658 |
| C11 | Defb1 | -1.0210 |
| C12 | Dennd4a | -1.1811 |
| D01 | Dffa | -1.2658 |
| D02 | Dpysl4 | -1.8532 |
| D03 | Eif5b | -1.1729 |
| D04 | Esr1 | -2.4795 |
| D05 | Fas | -1.4641 |
| D06 | Fasl | -1.8278 |
| D07 | Foxi1 | -1.1329 |
| D08 | Gaa | 1.257 |
| D09 | Gadd45a | -1.1811 |
| D10 | Galnt5 | -3.1162 |
| D11 | Grb2 | -1.1975 |
| D12 | Hspbap1 | 1.7532 |
| E01 | Htt | -1.1811 |
| E02 | Ifng | -1.1329 |
| E03 | Igf1 | -1.1173 |
| E04 | Igf1r | 1.0353 |
| E05 | Ins2 | 1.1329 |
| E06 | Irgm1 | -1.7292 |
| E07 | Jph3 | -1.6132 |
| E08 | Kcnip1 | -1.4539 |
| E09 | Mag | -1.4539 |
| E10 | Map1lc3a | -1.2311 |
| E11 | Mapk8 | 1.007 |
| E12 | Mcl1 | 1.2397 |
| F01 | Nfkb1 | -1.1408 |
| F02 | Nol3 | -1.9589 |
| F03 | Olfr1404 | -1.1329 |
| F04 | Parp1 | -1.2142 |
| F05 | Parp2 | -1.4142 |
| F06 | Pik3c3 | -1.0281 |
| F07 | Pvr | 1.879 |
| F08 | Rab25 | 1.3566 |
| F09 | Rps6kb1 | -1.3014 |
| F10 | S100a7a | -1.1329 |
| F11 | Snca | -1.3755 |
| F12 | Spata2 | -1.0792 |
| G01 | Sqstm1 | -1.0281 |
| G02 | Sycp2 | -1.8660 |
| G03 | Tmem57 | -1.4142 |
| G04 | Tnf | -1.1329 |
| G05 | Tnfrsf10b | -1.1019 |
| G06 | Tnfrsf11b | -1.6244 |
| G07 | Tnfrsf1a | 1.1975 |
| G08 | Traf2 | -1.0498 |
| G09 | Trp53 | -1.1811 |
| G10 | Txnl4b | 1.057 |
| G11 | Ulk1 | 1.0792 |
| G12 | Xiap | 1.007 |
| H01 | Actb | -1.3947 |
| H02 | B2m | -1.0644 |
| H03 | Gapdh | 1.0497 |
| H04 | Gusb | -1.0281 |
| H05 | Hsp90ab1 | 1.4845 |
| H06 | MGDC | -1.1329 |
| H07 | RTC | -1.3194 |
| H08 | RTC | -1.3287 |
| H09 | RTC | -1.3755 |
| H10 | PPC | -1.1729 |
| H11 | PPC | -1.1408 |
| H12 | PPC | -1.2311 |

**Supplementary Table 2.** PCR array dataset of the inferior colliculus (experiment 1), representing fold regulation in expression of the corresponding gene in the trauma group (noise exposed) compared to unexposed controls. Upregulated genes (fold regulation ≥ 2.0) are written in red, downregulated genes (fold regulation ≤ -2.0) are written in blue.

| **Position** | **Gene symbol** | **Fold regulation** |
| --- | --- | --- |
| A01 | 9430015G10Rik | -1.0046 |
| A02 | Abl1 | -1.1460 |
| A03 | Akt1 | -22.7273 |
| A04 | Apaf1 | -112.3596 |
| A05 | App | 1.0968 |
| A06 | Atg12 | 1.2002 |
| A07 | Atg16l1 | 1.0817 |
| A08 | Atg3 | -1.0400 |
| A09 | Atg5 | -1.0328 |
| A10 | Atg7 | -1.2541 |
| A11 | Atp6v1g2 | 1.1199 |
| A12 | Bax | -1.2369 |
| B01 | Bcl2 | -1.1147 |
| B02 | Bcl2a1a | -4.6168 |
| B03 | Bcl2l1 | -1.1382 |
| B04 | Bcl2l11 | 1.1837 |
| B05 | Becn1 | 1.0449 |
| B06 | Birc2 | -1.7250 |
| B07 | Birc3 | -1.5333 |
| B08 | Bmf | -1.9142 |
| B09 | Casp1 | -1.2628 |
| B10 | Casp2 | 1.1121 |
| B11 | Casp3 | -1.0046 |
| B12 | Casp6 | -4.2176 |
| C01 | Casp7 | -1.1303 |
| C02 | Casp9 | -1.8109 |
| C03 | Ccdc103 | -1.7013 |
| C04 | Cd40 | -1.2031 |
| C05 | Cd40lg | -1.4306 |
| C06 | Cflar | -1.3256 |
| C07 | Commd4 | -1.2455 |
| C08 | Ctsb | -1.0046 |
| C09 | Ctss | -1.0546 |
| C10 | Cyld | 1.1045 |
| C11 | Defb1 | -1.0400 |
| C12 | Dennd4a | -1.0046 |
| D01 | Dffa | 1.0521 |
| D02 | Dpysl4 | -1.4708 |
| D03 | Eif5b | -1.2716 |
| D04 | Esr1 | -1.1865 |
| D05 | Fas | -2.1683 |
| D06 | Fasl | 1.923 |
| D07 | Foxi1 | -1.4306 |
| D08 | Gaa | 1.1434 |
| D09 | Gadd45a | -1.1460 |
| D10 | Galnt5 | -6.8540 |
| D11 | Grb2 | -1.1783 |
| D12 | Hspbap1 | 2.9554 |
| E01 | Htt | -1.0918 |
| E02 | Ifng | -1.4708 |
| E03 | Igf1 | -2.1533 |
| E04 | Igf1r | -1.2628 |
| E05 | Ins2 | -3.2862 |
| E06 | Irgm1 | -1.4810 |
| E07 | Jph3 | -1.2716 |
| E08 | Kcnip1 | -1.0328 |
| E09 | Mag | -1.1541 |
| E10 | Map1lc3a | -1.2455 |
| E11 | Mapk8 | 1.1674 |
| E12 | Mcl1 | -1.1620 |
| F01 | Nfkb1 | -1.0400 |
| F02 | Nol3 | 1.192 |
| F03 | Olfr1404 | -1.4306 |
| F04 | Parp1 | -1.0842 |
| F05 | Parp2 | -1.1225 |
| F06 | Pik3c3 | -1.0693 |
| F07 | Pvr | 1.1355 |
| F08 | Rab25 | 1.0668 |
| F09 | Rps6kb1 | -1.2628 |
| F10 | S100a7a | -1.1147 |
| F11 | Snca | 1.0023 |
| F12 | Spata2 | -1.2804 |
| G01 | Sqstm1 | 1.0968 |
| G02 | Sycp2 | -4.7125 |
| G03 | Tmem57 | -129.8701 |
| G04 | Tnf | *-8.4388* |
| G05 | Tnfrsf10b | -1.2114 |
| G06 | Tnfrsf11b | -1.1620 |
| G07 | Tnfrsf1a | -1.8109 |
| G08 | Traf2 | 1.1277 |
| G09 | Trp53 | 1.0968 |
| G10 | Txnl4b | -1.0994 |
| G11 | Ulk1 | -1.0546 |
| G12 | Xiap | -1.3441 |
| H01 | Actb | -1.1541 |
| H02 | B2m | -1.1382 |
| H03 | Gapdh | N/A* |
| H04 | Gusb | -277.7778 |
| H05 | Hsp90ab1 | 1.3134 |
| H06 | MGDC | -1.4306 |
| H07 | RTC | -1.4405 |
| H08 | RTC | -1.5017 |
| H09 | RTC | -1.4110 |
| H10 | PPC | -1.4708 |
| H11 | PPC | -1.5333 |
| H12 | PPC | -1.4505 |

*Error. Other positive controls were used instead and consistently for other experiments.

**Supplementary Table 3.** PCR array dataset of the auditory cortex (experiment 1), representing fold regulation in mRNA expression of the corresponding gene in the trauma group (noise exposed) compared to unexposed controls. Upregulated genes (fold regulation ≥ 2.0) are written in red, downregulated genes (fold regulation ≤ -2.0) are written in blue.

| **Position** | **Gene symbol** | **Fold regulation** |
| --- | --- | --- |
| A01 | 9430015G10Rik | -1.2716 |
| A02 | Abl1 | 1.0234 |
| A03 | Akt1 | 1.0093 |
| A04 | Apaf1 | 1.192 |
| A05 | App | -1.3256 |
| A06 | Atg12 | -1.0918 |
| A07 | Atg16l1 | -1.1541 |
| A08 | Atg3 | -1.0116 |
| A09 | Atg5 | 1.234 |
| A10 | Atg7 | -1.0116 |
| A11 | Atp6v1g2 | -1.4810 |
| A12 | Bax | -1.1460 |
| B01 | Bcl2 | 1.1121 |
| B02 | Bcl2a1a | -6.7568 |
| B03 | Bcl2l1 | -1.1865 |
| B04 | Bcl2l11 | -1.3630 |
| B05 | Becn1 | -1.5017 |
| B06 | Birc2 | -1.5122 |
| B07 | Birc3 | -1.5333 |
| B08 | Bmf | -2.6151 |
| B09 | Casp1 | -1.2284 |
| B10 | Casp2 | 1.7211 |
| B11 | Casp3 | 1.0892 |
| B12 | Casp6 | 1.1045 |
| C01 | Casp7 | -1.0693 |
| C02 | Casp9 | -1.0116 |
| C03 | Ccdc103 | -1.3348 |
| C04 | Cd40 | -9.5602 |
| C05 | Cd40lg | 1.0234 |
| C06 | Cflar | 1.4373 |
| C07 | Commd4 | -1.6548 |
| C08 | Ctsb | -1.1783 |
| C09 | Ctss | -1.1460 |
| C10 | Cyld | -1.1303 |
| C11 | Defb1 | -3.6470 |
| C12 | Dennd4a | 1.1594 |
| D01 | Dffa | -1.0918 |
| D02 | Dpysl4 | -1.0046 |
| D03 | Eif5b | -1.2455 |
| D04 | Esr1 | -1.3441 |
| D05 | Fas | -3.9904 |
| D06 | Fasl | -3.6470 |
| D07 | Foxi1 | -3.0451 |
| D08 | Gaa | -1.0400 |
| D09 | Gadd45a | -1.2893 |
| D10 | Galnt5 | -1.0046 |
| D11 | Grb2 | -1.1225 |
| D12 | Hspbap1 | 3.2565 |
| E01 | Htt | 1.0521 |
| E02 | Ifng | 1.2687 |
| E03 | Igf1 | -1.6895 |
| E04 | Igf1r | -1.0994 |
| E05 | Ins2 | 1.1121 |
| E06 | Irgm1 | 1.2864 |
| E07 | Jph3 | -1.5439 |
| E08 | Kcnip1 | -1.3165 |
| E09 | Mag | -1.2369 |
| E10 | Map1lc3a | -1.3441 |
| E11 | Mapk8 | -1.1460 |
| E12 | Mcl1 | 1.1355 |
| F01 | Nfkb1 | 1.2426 |
| F02 | Nol3 | -1.0693 |
| F03 | Olfr1404 | -4.5517 |
| F04 | Parp1 | -1.2114 |
| F05 | Parp2 | -1.2716 |
| F06 | Pik3c3 | -1.3256 |
| F07 | Pvr | 1.1045 |
| F08 | Rab25 | 1.6625 |
| F09 | Rps6kb1 | -1.2284 |
| F10 | S100a7a | -1.4505 |
| F11 | Snca | -1.1071 |
| F12 | Spata2 | 1.0595 |
| G01 | Sqstm1 | -1.1071 |
| G02 | Sycp2 | -1.4011 |
| G03 | Tmem57 | 1.1514 |
| G04 | Tnf | -1.3165 |
| G05 | Tnfrsf10b | 1.8192 |
| G06 | Tnfrsf11b | 1.1837 |
| G07 | Tnfrsf1a | 1.0521 |
| G08 | Traf2 | 1.2255 |
| G09 | Trp53 | -1.0619 |
| G10 | Txnl4b | 1.3226 |
| G11 | Ulk1 | 1.3226 |
| G12 | Xiap | -1.1303 |
| H01 | Actb | -1.0257 |
| H02 | B2m | -1.0842 |
| H03 | Gapdh | 105240.708* |
| H04 | Gusb | -1.2984 |
| H05 | Hsp90ab1 | 1.1121 |
| H06 | MGDC | 1.1594 |
| H07 | RTC | -1.4011 |
| H08 | RTC | -1.3820 |
| H09 | RTC | -1.3630 |
| H10 | PPC | -1.7982 |
| H11 | PPC | -1.6895 |
| H12 | PPC | -1.6664 |

*Error. Other positive controls were used instead and consistently for other experiments.

**Supplementary Table 4.** PCR array dataset of the cochlear nucleus (experiment 2), representing fold regulation in mRNA expression of the corresponding gene in the trauma group (noise exposed) compared to unexposed controls. Upregulated genes (fold regulation ≥ 2.0) are written in red, downregulated genes (fold regulation ≤ -2.0) are written in blue.

| **Position** | **Gene symbol** | **Fold regulation** |
| --- | --- | --- |
| A01 | 9430015G10Rik | -1.3134 |
| A02 | Abl1 | 1.3348 |
| A03 | Akt1 | 1.162 |
| A04 | Apaf1 | -31.4139 |
| A05 | App | -1.0817 |
| A06 | Atg12 | 1.203 |
| A07 | Atg16l1 | -1.0668 |
| A08 | Atg3 | 1.0842 |
| A09 | Atg5 | 790.5247 |
| A10 | Atg7 | 1.0116 |
| A11 | Atp6v1g2 | -1.4077 |
| A12 | Bax | -1.0449 |
| B01 | Bcl2 | 1.3165 |
| B02 | Bcl2a1a | 6.9003 |
| B03 | Bcl2l1 | 1.0546 |
| B04 | Bcl2l11 | 120.8159 |
| B05 | Becn1 | -1.1514 |
| B06 | Birc2 | -1.0023 |
| B07 | Birc3 | 1.0918 |
| B08 | Bmf | 1.5984 |
| B09 | Casp1 | 2.9417 |
| B10 | Casp2 | -1.3044 |
| B11 | Casp3 | -1.4777 |
| B12 | Casp6 | 2.0801 |
| C01 | Casp7 | -1.0743 |
| C02 | Casp9 | 1.0994 |
| C03 | Ccdc103 | 1.0046 |
| C04 | Cd40 | -1.1837 |
| C05 | Cd40lg | 1.7983 |
| C06 | Cflar | 1.5874 |
| C07 | Commd4 | 1.0994 |
| C08 | Ctsb | 6920.5293 |
| C09 | Ctss | 1.6663 |
| C10 | Cyld | -1.5511 |
| C11 | Defb1 | -7.4815 |
| C12 | Dennd4a | -1.0968 |
| D01 | Dffa | 1.2114 |
| D02 | Dpysl4 | 153.9872 |
| D03 | Eif5b | 1102.5793 |
| D04 | Esr1 | -1.1121 |
| D05 | Fas | 1.4208 |
| D06 | Fasl | -1.8067 |
| D07 | Foxi1 | 1.7983 |
| D08 | Gaa | -1.4273 |
| D09 | Gadd45a | 1.203 |
| D10 | Galnt5 | -1.2086 |
| D11 | Grb2 | -1.0743 |
| D12 | Hspbap1 | 1.0767 |
| E01 | Htt | -1.0817 |
| E02 | Ifng | 1.7983 |
| E03 | Igf1 | 2.0946 |
| E04 | Igf1r | 1.0187 |
| E05 | Ins2 | 356.2304 |
| E06 | Irgm1 | 1.3074 |
| E07 | Jph3 | -1.4373 |
| E08 | Kcnip1 | -1.923 |
| E09 | Mag | 2.0373 |
| E10 | Map1lc3a | 1.2894 |
| E11 | Mapk8 | -1.2086 |
| E12 | Mcl1 | 1.0619 |
| F01 | Nfkb1 | 1.2983 |
| F02 | Nol3 | -1.1594 |
| F03 | Olfr1404 | 1.7983 |
| F04 | Parp1 | -1.0743 |
| F05 | Parp2 | -1.217 |
| F06 | Pik3c3 | 1.0187 |
| F07 | Pvr | 1.2541 |
| F08 | Rab25 | 16.0741 |
| F09 | Rps6kb1 | 1.0046 |
| F10 | S100a7a | 1.7859 |
| F11 | Snca | 1.6096 |
| F12 | Spata2 | 1.107 |
| G01 | Sqstm1 | -1.0023 |
| G02 | Sycp2 | -1.2255 |
| G03 | Tmem57 | -1.2426 |
| G04 | Tnf | 1.7983 |
| G05 | Tnfrsf10b | -1.0093 |
| G06 | Tnfrsf11b | -1.651 |
| G07 | Tnfrsf1a | 1.146 |
| G08 | Traf2 | 1.0767 |
| G09 | Trp53 | -1.0968 |
| G10 | Txnl4b | -1.0595 |
| G11 | Ulk1 | 1.0767 |
| G12 | Xiap | 1.0994 |
| H01 | Actb | 1.0473 |
| H02 | B2m | 1.0767 |
| H03 | Gapdh | -1.0968 |
| H04 | Gusb | 1.3535 |
| H05 | Hsp90ab1 | -1.1277 |
| H06 | MGDC | 1.7983 |
| H07 | RTC | 1.7983 |
| H08 | RTC | 1.7859 |
| H09 | RTC | 1.8361 |
| H10 | PPC | 1.8108 |
| H11 | PPC | 1.7613 |
| H12 | PPC | 1.9274 |

**Supplementary Table 5.** PCR array dataset of the inferior colliculus (experiment 2), representing fold regulation in mRNA expression of the corresponding gene in the trauma group (noise exposed) compared to unexposed controls. Upregulated genes (fold regulation ≥ 2.0) are written in red, downregulated genes (fold regulation ≤ -2.0) are written in blue.

| **Position** | **Gene symbol** | **Fold regulation** |
| --- | --- | --- |
| A01 | 9430015G10Rik | -1.3787 |
| A02 | Abl1 | -631.804 |
| A03 | Akt1 | 702.6521 |
| A04 | Apaf1 | 1.7013 |
| A05 | App | 1.0693 |
| A06 | Atg12 | 1.2283 |
| A07 | Atg16l1 | -1.192 |
| A08 | Atg3 | -1.0449 |
| A09 | Atg5 | -1.1674 |
| A10 | Atg7 | 128.5929 |
| A11 | Atp6v1g2 | 2.4396 |
| A12 | Bax | -1.2599 |
| B01 | Bcl2 | 1.0116 |
| B02 | Bcl2a1a | -8.0743 |
| B03 | Bcl2l1 | -1.5404 |
| B04 | Bcl2l11 | 94.7902 |
| B05 | Becn1 | 1.6434 |
| B06 | Birc2 | -1.2086 |
| B07 | Birc3 | -1.5511 |
| B08 | Bmf | -7.4299 |
| B09 | Casp1 | 4.0746 |
| B10 | Casp2 | 1.1947 |
| B11 | Casp3 | 1.4306 |
| B12 | Casp6 | -2.1189 |
| C01 | Casp7 | -1.2775 |
| C02 | Casp9 | -1.3597 |
| C03 | Ccdc103 | 1.2894 |
| C04 | Cd40 | -4.3873 |
| C05 | Cd40lg | -4.357 |
| C06 | Cflar | -2.4005 |
| C07 | Commd4 | -1.2864 |
| C08 | Ctsb | -1.6283 |
| C09 | Ctss | -1.6857 |
| C10 | Cyld | 1.8108 |
| C11 | Defb1 | -2.9759 |
| C12 | Dennd4a | 1.1381 |
| D01 | Dffa | -2.7959 |
| D02 | Dpysl4 | 1.1381 |
| D03 | Eif5b | 1.162 |
| D04 | Esr1 | -1.1199 |
| D05 | Fas | -3.3948 |
| D06 | Fasl | -1.7573 |
| D07 | Foxi1 | 2.3729 |
| D08 | Gaa | -1.3044 |
| D09 | Gadd45a | -2.1485 |
| D10 | Galnt5 | -30.5549 |
| D11 | Grb2 | -1.1277 |
| D12 | Hspbap1 | 2.7638 |
| E01 | Htt | -1.3318 |
| E02 | Ifng | -4.5106 |
| E03 | Igf1 | -2.3349 |
| E04 | Igf1r | 1.0116 |
| E05 | Ins2 | -65.4959 |
| E06 | Irgm1 | -1.7331 |
| E07 | Jph3 | 1.3074 |
| E08 | Kcnip1 | -1.3883 |
| E09 | Mag | 763.5965 |
| E10 | Map1lc3a | -1.341 |
| E11 | Mapk8 | 1.6548 |
| E12 | Mcl1 | -1.4777 |
| F01 | Nfkb1 | -1.977 |
| F02 | Nol3 | -1.0449 |
| F03 | Olfr1404 | -4.357 |
| F04 | Parp1 | 253.645 |
| F05 | Parp2 | 1.2283 |
| F06 | Pik3c3 | 1.0329 |
| F07 | Pvr | -1.2086 |
| F08 | Rab25 | -31.6324 |
| F09 | Rps6kb1 | 1.0767 |
| F10 | S100a7a | 3.4983 |
| F11 | Snca | 7.7633 |
| F12 | Spata2 | 1.4914 |
| G01 | Sqstm1 | -1.2002 |
| G02 | Sycp2 | 1.6896 |
| G03 | Tmem57 | 483.2636 |
| G04 | Tnf | -1.8575 |
| G05 | Tnfrsf10b | -1.4077 |
| G06 | Tnfrsf11b | -1.2775 |
| G07 | Tnfrsf1a | 92.8395 |
| G08 | Traf2 | -1.1755 |
| G09 | Trp53 | -1.1514 |
| G10 | Txnl4b | -1.1045 |
| G11 | Ulk1 | -1.6396 |
| G12 | Xiap | -1.8575 |
| H01 | Actb | -1.3692 |
| H02 | B2m | 1.0401 |
| H03 | Gapdh | -1.2426 |
| H04 | Gusb | -1.2953 |
| H05 | Hsp90ab1 | 1.3165 |
| H06 | MGDC | -4.357 |
| H07 | RTC | -4.0093 |
| H08 | RTC | -4.7349 |
| H09 | RTC | -4.5736 |
| H10 | PPC | -5.4014 |
| H11 | PPC | -3.302 |
| H12 | PPC | -4.7349 |

**Supplementary Table 6.** PCR array dataset of the auditory cortex (experiment 2), representing fold regulation in mRNA expression of the corresponding gene in the trauma group (noise exposed) compared to unexposed controls. Upregulated genes (fold regulation ≥ 2.0) are written in red, downregulated genes (fold regulation ≤ -2.0) are written in blue.

| **Position** | **Gene symbol** | **Fold Regulation** |
| --- | --- | --- |
| A01 | 9430015G10Rik | 1.2716 |
| A02 | Abl1 | 1.6434 |
| A03 | Akt1 | 1.4607 |
| A04 | Apaf1 | 1.0116 |
| A05 | App | 1.0693 |
| A06 | Atg12 | -1.0377 |
| A07 | Atg16l1 | 1.1865 |
| A08 | Atg3 | 1.0257 |
| A09 | Atg5 | 1.3724 |
| A10 | Atg7 | -1.0595 |
| A11 | Atp6v1g2 | -1.2599 |
| A12 | Bax | 1.3724 |
| B01 | Bcl2 | 1.0693 |
| B02 | Bcl2a1a | 15.5266 |
| B03 | Bcl2l1 | 4597.6045 |
| B04 | Bcl2l11 | -1.192 |
| B05 | Becn1 | -1.1674 |
| B06 | Birc2 | 986.8361 |
| B07 | Birc3 | 1.5122 |
| B08 | Bmf | 2.9828 |
| B09 | Casp1 | -5.9518 |
| B10 | Casp2 | 1.6663 |
| B11 | Casp3 | 1.6663 |
| B12 | Casp6 | 1.2198 |
| C01 | Casp7 | 1.3256 |
| C02 | Casp9 | 1.203 |
| C03 | Ccdc103 | -2.1634 |
| C04 | Cd40 | 3.6723 |
| C05 | Cd40lg | 2.5609 |
| C06 | Cflar | 1.3074 |
| C07 | Commd4 | 1.5227 |
| C08 | Ctsb | 1.7132 |
| C09 | Ctss | 1.0619 |
| C10 | Cyld | 1.1865 |
| C11 | Defb1 | 7.1437 |
| C12 | Dennd4a | 1.107 |
| D01 | Dffa | 1.5764 |
| D02 | Dpysl4 | 1.2114 |
| D03 | Eif5b | 1.0994 |
| D04 | Esr1 | 1.1381 |
| D05 | Fas | 2.1092 |
| D06 | Fasl | 2.1535 |
| D07 | Foxi1 | -3.0175 |
| D08 | Gaa | 2.1238 |
| D09 | Gadd45a | 1.4506 |
| D10 | Galnt5 | 2.9622 |
| D11 | Grb2 | 1.2983 |
| D12 | Hspbap1 | 1.4914 |
| E01 | Htt | 1.0046 |
| E02 | Ifng | 4.6161 |
| E03 | Igf1 | 1.3074 |
| E04 | Igf1r | -1.0305 |
| E05 | Ins2 | -5.2902 |
| E06 | Irgm1 | 1.2368 |
| E07 | Jph3 | -1.1434 |
| E08 | Kcnip1 | 2.8812 |
| E09 | Mag | 4.2183 |
| E10 | Map1lc3a | 1.146 |
| E11 | Mapk8 | -1.3692 |
| E12 | Mcl1 | 1.5547 |
| F01 | Nfkb1 | 1.9816 |
| F02 | Nol3 | 1.2454 |
| F03 | Olfr1404 | 2.5609 |
| F04 | Parp1 | 1.0842 |
| F05 | Parp2 | 1.3348 |
| F06 | Pik3c3 | 1.2368 |
| F07 | Pvr | -1.0093 |
| F08 | Rab25 | 1.9954 |
| F09 | Rps6kb1 | 1.0918 |
| F10 | S100a7a | -2.1337 |
| F11 | Snca | -6.8369 |
| F12 | Spata2 | -1.1594 |
| G01 | Sqstm1 | 1.3256 |
| G02 | Sycp2 | 1.7859 |
| G03 | Tmem57 | 1.2368 |
| G04 | Tnf | -1.8834 |
| G05 | Tnfrsf10b | 1.8877 |
| G06 | Tnfrsf11b | 1.8877 |
| G07 | Tnfrsf1a | 1.9009 |
| G08 | Traf2 | 1.5333 |
| G09 | Trp53 | 1.4109 |
| G10 | Txnl4b | 1.0994 |
| G11 | Ulk1 | 1.4506 |
| G12 | Xiap | 1.6663 |
| H01 | Actb | 1.1947 |
| H02 | B2m | -1.0668 |
| H03 | Gapdh | 1.2454 |
| H04 | Gusb | 1.4109 |
| H05 | Hsp90ab1 | -1.1199 |
| H06 | MGDC | 2.5609 |
| H07 | RTC | 2.8415 |
| H08 | RTC | 2.7258 |
| H09 | RTC | 2.7638 |
| H10 | PPC | 3.1748 |
| H11 | PPC | 2.9622 |
| H12 | PPC | 3.1529 |

**Supplementary Figure**

**
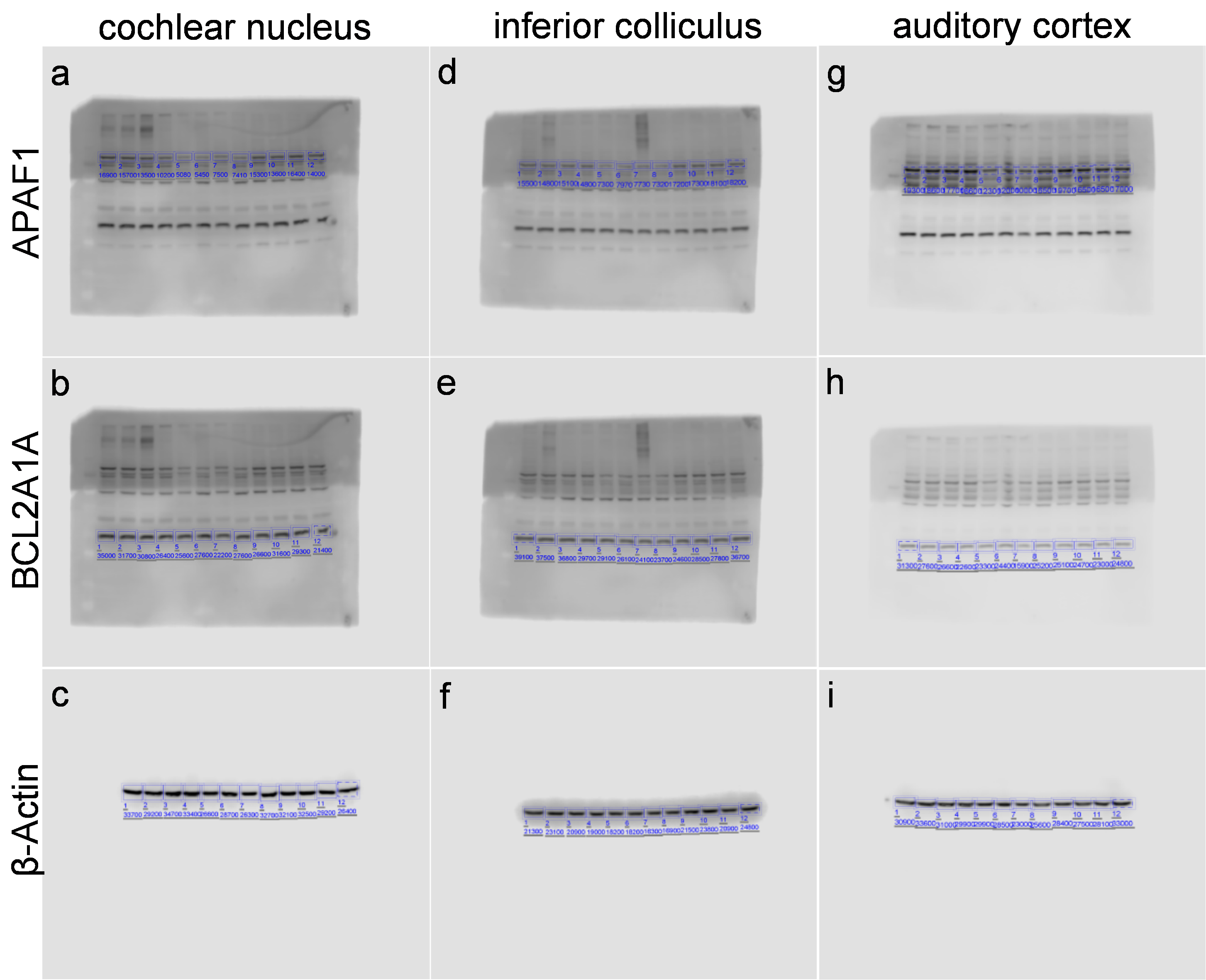
**

**Supplementary Figure 1.** Original images of western blot membranes from protein detection experiments. Western blot bands of the two measured proteins (a,d,g: APAF1; b,e,h: BCL2A1A) are shown for each investigated structure (left: cochlear nucleus; middle: inferior colliculus; right: auditory cortex). Dashed rectangles indicate areas of protein signal chemilumenescence measurements. Data was normalized to surrounding background chemilumenescence signal intensity (measurement area indicated by larger solid rectangles (surrounding dashed rectangles)) for each protein sample, respectively (n=12). Numbers below show quantified relative protein signal intensities. Beta-Actin (c,f,i) acts as house keeping protein and was used as a reference for standardization of measured APAF1 and BCL2A1A signal intensity for each structure.
